# Novel Local Chimeric Flap Based on Tunnelized Facial Artery Myomucosal Island Flap and Submandibular Gland Flap for Reconstructions After Oral Squamous Cell Carcinoma Surgery

**DOI:** 10.1097/SCS.0000000000008862

**Published:** 2022-08-09

**Authors:** Lukas Hauer, Petr Posta, Jiri Gencur, Christos Micopulos, Jan Liska, Jaroslava Podesvova, Kristyna Pivovarcikova, Omid Moztarzadeh

**Affiliations:** * Department of Stomatology, University Hospital Pilsen, Faculty of Medicine in Pilsen, Charles University; † Department of Anaesthesiology and Intensive Care Medicine, University Hospital Pilsen, Faculty of Medicine in Pilsen, Charles University; ‡ Sikl’s Department of Pathology, Faculty of Medicine in Pilsen, Charles University; § Bioptic Laboratory Ltd; ∥ Department of Anatomy, Faculty of Medicine in Pilsen, Charles University, Pilsen, Czech Republic

**Keywords:** oral squamous cell carcinoma, reconstructive surgery, t-FAMMIF-SMG local chimeric flap

## Abstract

The reconstruction of oral tongue and floor of mouth defects after resections of squamous cell carcinoma is a challenging task in reconstructive surgery aiming for appropriate restoration of oral function and quality of life improvement. In this study, the authors introduce the innovative reconstruction technique of medium-sized defects consisting of tunnelized facial artery myomucosal island flap and submandibular gland flap as the local chimeric flap pedicled on facial vessels. A retrospective case series evaluation of 4 patients suffering from oral cavity cancer (stages III and IVa), who underwent transoral tumor excision with neck dissection and immediate reconstruction in the time period September 2020 to July 2021, was conducted. No flap losses or flap-related complications were identified. No recurrences occurred during the follow-up at 11.0±4.5 months (range: 6–16 mo, median=11 mo). Tunnelized facial artery myomucosal island flap and submandibular gland flap local chimeric flap expands the reconstruction options of medium-sized defects after ablative oral cancer surgery in carefully selected patients primarily not suitable for free flap reconstructions.

Squamous cell carcinoma (SCC) of the oral tongue and floor of the mouth is the most commonly diagnosed type of oral cancer.^1^ The multidisciplinary approach is necessary for planning personalized treatment strategies taking into consideration detailed characteristics of both the tumor and the patient. If possible, a radical resection with adequate margins and an appropriate type of neck dissection is currently preferred as the first-choice treatment.^1^,^2^ An adjuvant oncological therapy is indicated to achieve better local control and prognosis improvement according to the disease stage and existing pathological risk factors.

For the essential attainment of microscopically clear margins (>5 mm), additional excision of healthy tissue (>10–15 mm) from the gross tumor border is mandatory during the ablative surgery.^3^ Therefore, resulting defects often require adequate replacement of lost soft tissues to establish form and function in the resected organ even in cases of early-stage tumors. The aim is to prevent tongue fixation accompanied by limited function in terms of correct mastication, swallowing, and speech. This is rarely achieved by a primary wound closure or leaving defects to spontaneous epithelialization. To provide satisfactory restoration of oral function and facial aesthetics, reconstruction techniques are chosen according to various local and systemic factors such as the defect size and localization, general and individual anatomical features, tissue match, compliance and overall health of the patient and last but not least the surgical expertise. The current trend is to perform a microvascular oral tongue and floor of mouth reconstruction, which is indispensable in cases of complex and/or large defects.^2^,^4^ But usually applied free flaps (eg, a radial forearm, lateral arm, or anterolateral thigh perforator flap)^4^,^5^ may not be suitable for medium-sized defects (too bulky) and in comorbid patients with poor surgical tolerance and need for a shortened procedure.^3^,^6^ For these instances, various local or locoregional flaps may be used. Among these, facial artery myomucosal island flap (t-FAMMIF) or submandibular gland (SMG) flap have previously been proven as versatile, reliable, well-vascularized, and easy to harvest flaps enabling replacement with a similar kind of tissue. From the aesthetic point of view, the donor site of these flaps is hidden or does not create the necessity for an additional external approach in patients undergoing neck dissection, in addition, it is associated with minimal morbidity.^3^,^7^–^12^

The long-term clinical experience with both flaps, the current detailed knowledge of their harvesting technique and the precise anatomy of vascular supply enable to newly design local chimeric flap pedicled on the same facial vessels and consisted of the above-mentioned flap combination. The chimeric flap concept described by Hallock^13^ and refined by Agarwal et al^14^ is characterized by a flap consisting of multiple tissue components of varied tissue types supplied by separate vascular branches which are attached to a single source vessel and allow for spatially independent tissue placement. This type of reconstructive technique originally proposed as microvascular free tissue transfer is suitable especially for extensive, complex and 3-dimensional defects and can provide a 1-stage reconstruction with less effort and operation time.^15^ Tunnelized facial artery myomucosal island flap and SMG chimeric flap meets the criteria for the chimeric flap, but it is the local flap which due to its pedicle formed by skeletonized vessels has pedicle-related advantages of the free flap (1-stage procedure without sectioning of buried pedicle, wide arc of rotation) without need for the microvascular anastomosis. Tunnelized facial artery myomucosal island flap and SMG local chimeric flap, by its nature, provides a relatively large amount of tissue for reconstruction, namely t-FAMMIF for mucosal defects replacement and SMG for filling deeper dead space or even a surface restoration when placed posteriorly to t-FAMMIF. If used in a cancer setting, careful patients selection for this reconstructive procedure is necessary to ensure oncological safety, primarily with regard to a cervical lymph node status. This study aims at evaluating a retrospective case series of 4 patients suffering from oral tongue and floor of mouth SCC, who underwent previously unreported, innovative reconstruction technique with t-FAMMIF-SMG local chimeric flap. Assessment of surgical protocol, success rate, safety and clinical effects of this reconstruction method was conducted.

## MATERIALS AND METHODS

The evaluation of single-institution, retrospective consecutive case series was performed. All patients suffering from oral tongue and floor of mouth SCC, who underwent transoral tumor resection, neck dissection, and immediate reconstruction with t-FAMMIF-SMG local chimeric flap in the period September 2020 to July 2021 were included. The inclusion criteria for this type of reconstruction were: patient without previous surgery or radiotherapy to the area of interest, clinical-stage T2–T3, cN0—minimally in levels I to II ipsilateral to the flap without obvious ipsilateral extranodal extension on the neck based on the whole-body fluorine-18 fluorodeoxyglucose positron emission tomography (PET)/computed tomography or PET/magnetic resonance imaging staging, absence of chronic mucosal lesion on the buccal mucosa planned for harvesting, presumed medium-sized defect after ablative surgery, need for less time-consuming surgery due to advanced biological age and/or comorbidities. The following parameters were monitored and evaluated in the study group: correlation between clinical and pathological stage of the disease according to eighth edition of the Union for International Cancer Control TNM classification,^16^ types of ablative surgery, wound healing complications in the recipient or donor site, flap-related complications, adjuvant therapy, oncological outcomes, follow-up, and functional and cosmetic results as described by Ferrari et al^10^ (mouth opening—0 serious limitations<0.5 cm, 1 partial limitation 0.5–1.5 cm, 2 minimal limitations 1.5–3.0 cm, 3 no limitation, oral commissure symmetry—0 severe downward retractions of oral commissure with severe asymmetry, 1 moderate retraction and asymmetry, 2 mild alterations of symmetry, 3 complete symmetry preservation) and oral intake level after surgery (0—none, full percutaneous endoscopic gastrostomy dependence, 1 partial, peroral soft diet combined with nutrition via gastrostomy, 2 full oral intakes).

### Surgical Procedure Protocol

The tumor in the oral cavity was first resected via transoral approach according to generally accepted principles of oral cancer surgery (an en bloc resection with adequate surgical margins of >1 cm). The defect size was then assessed in terms of planned reconstruction feasibility. The skin neck incision was performed depending on the type of neck dissection, which was further carried out in a standard manner focusing on the following aspects: In cases of bilateral neck dissections t-FAMMIF-SMG local chimeric flap was harvested on the less risky side regarding oncological safety (see the inclusion criteria). The SMG was preserved as a part of the chimeric flap pediceled inferiorly on facial vessels. The venous drainage of the flap is provided by the venae comitantes of the facial artery and anterior facial vein draining into the internal or external jugular vein. The arterial blood supply is given by the facial artery, the external carotid artery branch. Harvesting of the flap was started with the dissection of the distal venous drainage due to a frequent manifestation of diverse anatomical variabilities. Finding the drainage course of the facial vein was crucial in vascular pedicle dissection. Releasing of SMG as a part of chimeric flap resembles the conventional SMG extirpation performed in an extracapsular plane with the precise clearance of all fibrofatty and lymphatic tissue in neck levels I to II. Preservation of main glandular branches derived from the facial artery as well as accessory branches commenced mainly from the submental artery is mandatory. The same approach is necessary for the venous system (glandular branches are also predominantly drained into the facial and submental vein). For the increase of the vascular pedicle length and arc of flap rotation, the main trunks of facial vessels were dissected from their origin (artery) or end (vein) up to the lower border of the mandible. Here, the marginal mandibular branch of the facial nerve was identified and freed from surrounding tissue to allow the pull-through maneuver of t-FAMMIF. Submental vessels were ligated and transected at the anterosuperior border of the gland. The entire SMG as a part of the chimeric flap was released from the submandibular space by ligation and transection of the posterior facial vein, Wharton duct, hilar vessels, and by transection of submandibular ganglion, freeing the flap from the lingual nerve. Further, the facial vessels were dissected at the level above the marginal mandibular branch of the facial nerve creating an inferior part of the tunnel into the cheek. Harvesting t-FAMMIF in the oral cavity was performed as described by Massarelli et al^9^ which includes the buccal artery angiosome. However, great care was required to avoid the buccal fat pad disruption. The t-FAMMIF transposition to the neck area under the marginal mandibular branch of the facial nerve was the next step with caution to maintain the nerve integrity. The entire t-FAMMIF-SMG local chimeric flap inferiorly pediceled on skeletonized facial vessels was then turning around the inferior mandibular border and transposed into the oral surgical defect routing the pedicle behind the dorsal edge of mylohyoid muscle or through a sufficient artificial tunnel in the mouth floor. A vascular pedicle twisting was avoided by proper flap placement and its suture fixation. t-FAMMIF was available for surface reconstruction and SMG for filling deeper defects or even the mucosal reconstruction when positioned posteriorly to t-FAMMIF. To avoid retraction and fibrosis, the cheek donor site was draped using the buccal fat pad sutured to defect margins. The healing at the donor site and on SMG exposed to the oral cavity (if used) was by granulation tissue formation with subsequent epithelialization. In the postoperative period, all patients received the prophylactic dosage of low–molecular-weight heparin not only to prevent pedicle thrombosis, and all were fed via percutaneous endoscopic gastrostomy inserted preoperatively.

## RESULTS

**FIGURE 1:**
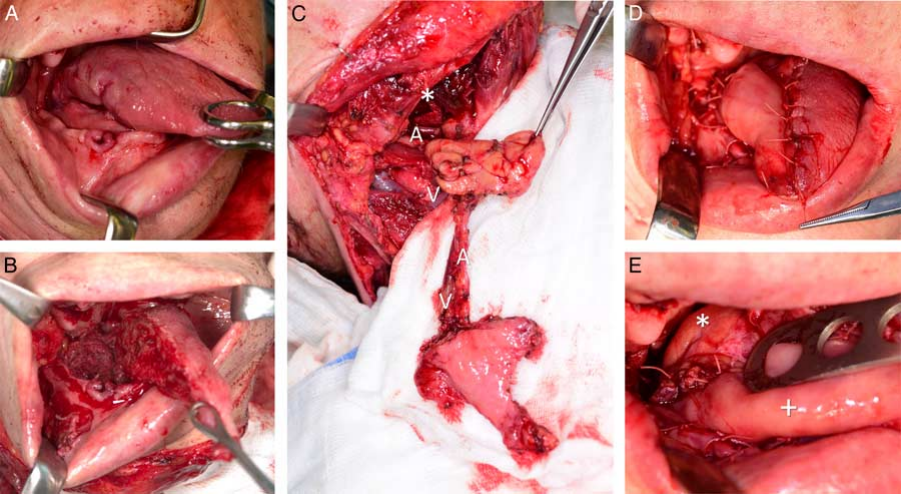
Patient no. 3. The endophytic squamous cell carcinoma of the right tongue margin reaching the midline, state after extraction of tooth 43. (A) State after extended hemiglossectomy and harvesting of the right t-FAMMIF-SMG local chimeric flap, a temporary hypopharyngeal pack is present. (B) Tunnelized facial artery myomucosal island flap and submandibular gland local chimeric flap before transposition into the oral surgical defect. (C) Marginal mandibular branch of facial nerve freed and running behind the mandibular angle (*). State immediately after the reconstruction, submandibular gland was placed posteriorly to tunnelized facial artery myomucosal island flap not only for surface restoration. (D) The close-up view of reconstructed tongue root. Submandibular gland (*)/tunnelized facial artery myomucosal island flap (+) as a part of local chimeric flap. (E) A/V indicates artery/vein of the flap pedicle.

**FIGURE 2:**
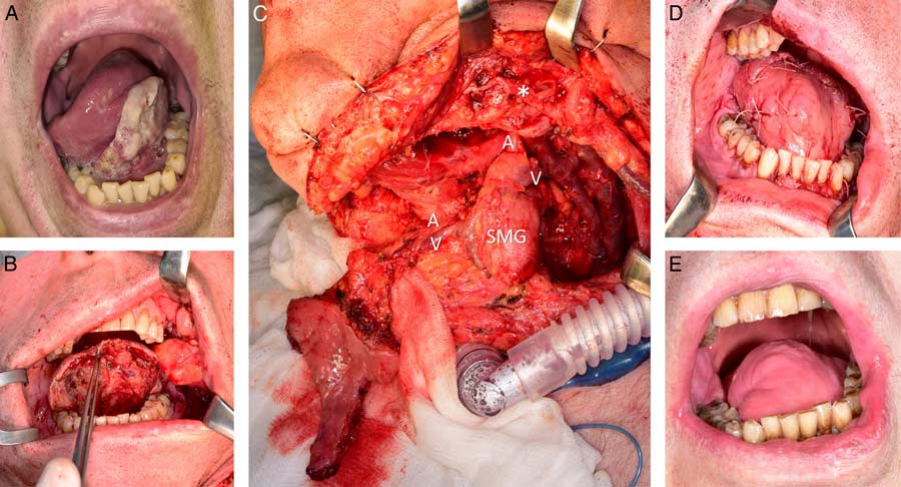
Patient no. 4. Squamous cell carcinoma of tongue ventral surface and floor of the mouth. (A) State after tumor excision with extended anterior hemiglossectomy and harvesting of the left tunnelized facial artery myomucosal island flap and SMG local chimeric flap, the intact buccal fat pad, and cuff of lower vestibule mucosa for smooth donor site closure is well visible. (B) Tunnelized facial artery myomucosal island flap and SMG local chimeric flap before transposition into the oral surgical defect. Present variations of deep venous system may be further simplified for increasing flap rotation arc. (C) Marginal mandibular branch of facial nerve. (*). State immediately after the reconstruction, SMG was used for filling dead space and covered by tunnelized facial artery myomucosal island flap. (D) State 6 months after surgery and 2 months after adjuvant chemoradiotherapy. (E) A/V indicates artery/vein of the flap pedicle; SMG, submandibular gland.

**FIGURE 3:**
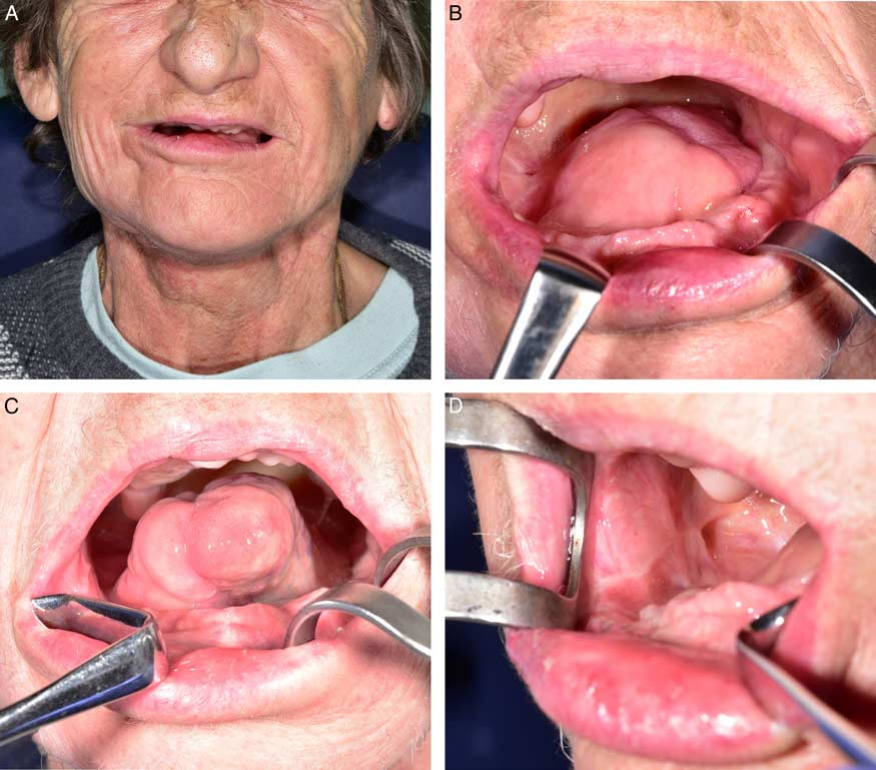
Functional and aesthetic outcomes of the patient no. 3 seven months after surgery. Extraoral view (A), intraoral view with resting position of tongue (B), tongue protrusion (C), right cheek, the donor site of tunnelized facial artery myomucosal island flap part of local chimeric flap (D).

In total, 4 patients met the inclusion criteria and were enrolled in this study. A shorter surgery duration requirement and/or unsuitable conditions for microvascular reconstruction were present in all of these patients mainly due to: abdominal aorta and iliac arteries atherosclerosis (no. 1), advanced age, state after lower extremity deep vein thrombosis and hypothyroidism (no. 2), chronic obstructive pulmonary disease, fatty liver disease, atrial fibrillation, peripheral arterial disease, arterial hypertension, state after lower extremity deep vein thrombosis, severe thoracic kyphoscoliosis, osteoporosis (no. 3), severe chronic obstructive pulmonary disease, arterial hypertension, state after COVID-19, state after the evacuation of chronic subdural hematoma with subsequent epileptic seizure (no. 4). Demographic data, tumor characteristics including clinical and pathological stage, the type of ablative surgery, and the side localization of t-FAMMIF-SMG local chimeric flap, local postoperative complications, adjuvant therapy, oncological outcomes, and follow-up durations are summarized in Supplementary Digital Content (Table 1, http://links.lww.com/SCS/E296) and Figure 1 and 2. The male-to-female ratio was 1:1. The mean age of patients was 66.0±8.1 years (range: 57–75 y, median=66 y). The SMG as a part of the chimeric flap was used for mouth diaphragm reconstruction in 2 cases (nos 1 and 2), once for dead space-filling (no. 4) and once for surface restoration by positioning posteriorly to t-FAMMIF (no. 3). Although the time required for flap harvesting was not measured accurately, because harvesting essentially begins during neck dissection, the total time did not usually exceed 1 hour. All flaps were successful and no flap losses or flap-related complications were identified. Wound healing complications were detected in 2 patients. Patient no. 2 developed a residual tongue partial necrosis 8 days after the procedure. The necrectomy and tongue stump resuturing to the flap was performed under local anesthesia. One month after the primary surgery, a mandibular fracture occurred in edentulous area 33 managed with an open reduction from external approach and internal load-bearing fixation by plate and screws. Further healing was uneventful. In patient no. 4, surgical site infection emerged a few days after the surgery, resolved after antiseptic rinses and systemic antibiotic therapy adjusted to antimicrobial susceptibility testing results. Only 1 patient (no. 3) did not undergo adjuvant cancer therapy as a consequence of severe thoracic kyphoscoliosis causing irradiation position intolerance. Functional and aesthetic outcomes are summarized in Supplementary Digital Content (Table 2, http://links.lww.com/SCS/E296) and Figure 3. No case of permanent facial nerve palsy was detected. The follow-up duration was 11.0±4.5 months (range: 6–16 mo, median=11 mo).

## DISCUSSION

The current state of the art for tongue and floor of mouth reconstruction is focused on maintaining tongue mobility, especially its protrusion and elevation to provide adequate speech and food control.^4^ But to date, still no reconstruction technique is available for proper replacement of coordinate tongue movement. Any flap used supplies only a lost tissue volume, and the mobility is ensured by the residual tongue. So, a better functional outcome is usually achieved in cases of more tongue musculature preservation (if possible) and where the tongue remnant movement is facilitated by the appropriate reconstruction method. Moreover, the recreating of the floor of the mouth diaphragm is required to avoid loss of flap position (inferior displacement) in cases of extensive ablative surgery.^4^

Tunnelized facial artery myomucosal island flap was first described by Zhao et al^7^ as a buccinator myomucosal island flap used for partial tongue reconstruction in 2003. Later, Massarelli and colleagues^9^,^17^ specified in detail and improved its surgical technique. To date, various types of buccinator myomucosal flaps have been widely used for tongue and floor of mouth reconstruction.^3^,^18^–^20^ Due to the insufficient terminology standardization and inaccurate, even conflicting nomenclature the new, simplified classification of buccinator myomucosal flaps was proposed in 2017.^21^ The SMG flap was first introduced by Zietek and colleagues^22^,^23^ for hypopharyngeal and laryngeal reconstructions in 1998 and 1999, respectively. Since that time, it has been used for oral, oropharyngeal, infratemporal, and parotidectomy defects repair.^11^,^12^,^24^,^25^ Submandibular gland flap used in reconstructions after ablative surgery for oral SCC may be perceived controversially, because of its oncological safety. Three different types of SMG involvement by oral SCC have been recognized: direct primary tumor invasion, involvement by extranodal extension of level Ib/IIa lymph node metastasis, and the frank intraglandular lymph node metastasis. Unlike the parotid gland, the presence of lymph nodes in SMG has still not been fully clarified. Submandibular gland metastases associated with intraglandular nodes are exceptionally rare, and the probability of these types of SMG involvement is almost negligible.^12^ If the SMG metastasis occurs, most often it is a hematogenous metastasis originating from a distant organ. Recently, Zeng et al^12^ summarized and analyzed data dealing with 2875 SMG samples from 2750 patients suffering from oral and oropharyngeal cancer, where 59 (2.05%) SMG was involved, including 44 samples of direct primary tumor invasion, 13 of involvement by extranodal extension, and only 2 of frank intraglandular lymph node metastasis. Based on these findings, it can be stated that the SMG preservation in cases without obvious direct primary tumor or extranodal invasion is oncologically safe.

**FIGURE 4:**
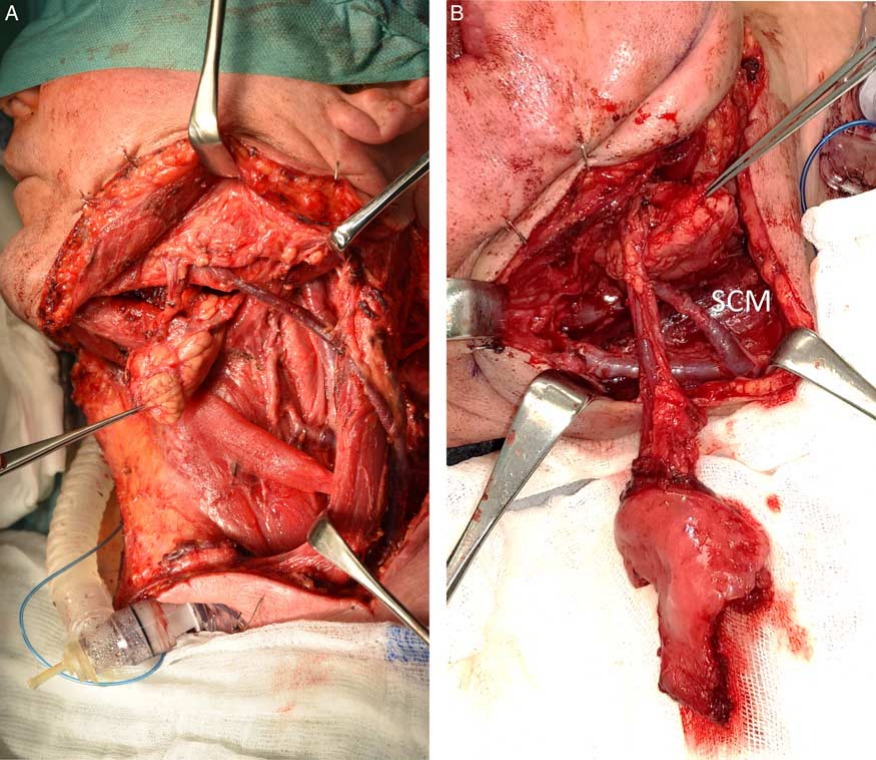
Venous system variability. A patient not included in present study with facial vein drained into external jugular vein but without venous branches from submandibular gland. For ensuring the submandibular gland venous drainage the preservation of concomitant Wharton duct vein is mandatory in these cases (A), The right tunnelized facial artery myomucosal island flap and submandibular gland local chimeric flap with the venous pedicle drained into external jugular vein in patient no. 1 (B). SCM indicates sternocleidomastoid muscle.

The t-FAMMIF-SMG flap is a local, anterograde branch-based chimeric flap formed by the part of buccinator and also orbicularis oris muscle, submucosa with minor salivary glands, and the buccal mucosa as one flap component with the entire SMG as the remaining part of the flap. For increasing the mucosal paddle or the muscle bulk of the flap the parotid duct transposition or incision in different planes in the lower vestibule (buccal mucosa at a higher level than buccinator muscle) is recommended.^17^ The vascular supply of this inferiorly pediceled flap is provided by skeletonized facial vessels. The nourishing branches for the flap part composed of t-FAMMIF are: posterior buccal branch anastomosed with the buccal artery (branch of the second portion of the maxillary artery), 1 to 3 inferior buccal branches, and 3 to 5 anterior buccal branches, all of them originate from the facial artery.^7^ The inclusion of the neighboring buccal artery angiosome into the flap is required. After flap harvesting, it enables good vascularization up to the dorsal periphery via the reverse blood flow in the direction from facial to buccal artery in a manner similar to choke arteries.^9^ Venous blood of this part of the flap is collected by a few veins forming buccal venous plexus on the dorsal portion of the muscle and draining into the facial vein from 2 to 4 tributaries.^7^ The facial vein maybe sometimes rudimentary, running very close to the artery. Careful dissection with its preservation and including it within the flap is mandatory in these cases. The SMG in the flap is supplied by 2 to 5 arterial branches. The main nourishing branches are derived from the facial artery and the accessory ones from the submental artery. Venous drainage is provided by 2 to 6 veins, whereas the main branches are drained into the facial vein and the accessory ones into the submental vein.^26^ The most constant main venous branch accompanying the Wharton duct and draining into the sublingual vein (concomitant Wharton duct vein)^26^ have to be sacrificed for the appropriate rotation arc and the spatial freedom for flap insetting. Its preservation is mandatory only on rare occasions when it is the only main vein providing the SMG venous drainage, thereafter not fulfilling the criteria for the local chimeric flap (Fig. 4A). The flap pedicle length is usually sufficient for the contralateral side reconstruction giving an advantage in terms of oncological safety. According to cadaveric studies, the average distance from facial artery origin to its first SMG branch was 32 mm, and to submental artery origin, it was 46 mm.^26^ The mean distance from the lower part of the mandibular inferior border to the beginning of the t-FAMMIF mucosal paddle was 48 to 49 mm.^27^ Primarily, high variability of the neck venous system may become a limitation not only for the feasibility of flap harvesting but also for maneuvering and insetting the flap due to its pedicle length. Findings such as facial vein drained directly into external jugular vein, or other venous drainage anomalies are not uncommon.^28^ In 1 of 4 of our patients (no. 1) the drainage of the facial vein into the external jugular vein was also revealed (Fig. 4B). In the present case series, the SMG was used as a deeper defect filling or mouth diaphragm reconstruction in 3 patients, and in 1 case the SMG also repaired an oral lining. The complete epithelialization within 3 to 6 weeks has been reported with this use, similar to the epithelialization of buccal fat pad (within 3–4 wk) at the donor site.^10^–^12^ The same results were also observed in our study. In the year 2014, Zhang et al^6^ reported case series of 8 elderly patients with various comorbidities who underwent oral defects reconstruction after SCC ablative surgery by superiorly pediceled sternocleidomastoid myocutaneous flap (mouth diaphragm repair) and inferiorly pediceled SMG flap (oral mucosal defects repair). In the year 2019, Zeng et al^12^ reported 1 case out of 15 reconstructions with SMG flap, where submental island pedicled flap combined with SMG flap was used to repair postoperative oral SCC defect of the gingival site and partial mandible. Compared with these studies, t-FAMMIF-SMG local chimeric flap offers replacement of mucosa by the same kind of tissue with the required bulk provided by the SMG part of the flap, in addition with only 1 mother pedicle supply. The donor site morbidity of the t-FAMMIF-SMG local chimeric flap is mainly related to t-FAMMIF harvesting including limitation in mouth opening, mandibular vestibule obliteration, parotid duct, or facial nerve injury with the resulting aesthetics or functional consequences. If donor site management follows the principles established by Ferrari et al^10^ (harvesting in different planes in the lower vestibule, closure with buccal fat pad advancement, postoperative physiotherapy, and cheek massages), the morbidity is extremely low. All complications in our patients (surgical site infection, partial necrosis of residual tongue, mandibular fracture) occurred in the recipient site but were mostly related to the extent of ablative surgery, not the reconstructive surgical procedure. If a tumor occurred in the floor of the mouth causes the Wharton duct obstruction, inflammatory changes in the SMG may make it unsuitable for reconstruction, and the contralateral side should be a better option. Functional outcomes in terms of oral intake, mouth opening, and even facial aesthetics were satisfactory in our case series. These results should be viewed in the light of a short period from surgery and primarily adjuvant therapy, significantly affecting mouth opening. The patients condition has been still improving so the effect at the time of writing this manuscript cannot be considered definitive.

The oncological safety of t-FAMMIF-SMG local chimeric flap may be based on the current state of knowledge using these involved flaps independently. According to this, the facial artery preservation during elective neck dissection in clinical N0 cervical lymph node status does not compromise oncological safety.^29^ However, in cases of clinical-stage N+ with a therapeutic neck dissection requirement, the significance of preserving facial artery in relation to oncological safety has not yet been established.^3^ With regard to technical aspects, cervical lymph node clearance around SMG and relatively large caliber vessels of the pedicle is well feasible. Due to the fact that in a number of studies pathological staging in previously stated clinically N0 neck has detected the occult metastases occurrence even at the level I without oncological safety impairment,^3^,^8^,^12^,^29^ the main problem does not appear to be in the presence of lymph node metastases as such, but the manifestation of extranodal extension. In our case series, 3 patients underwent reconstruction by a t-FAMMIF-SMG local chimeric flap from the contralateral side to lymph nodes suspicious for metastasis (nos 1 and 4) or contralaterally to primary tumor deeply invading the floor of the mouth (no. 2). The same approach was also published by other authors.^7^,^8^ In 1 case (no. 3), the flap was harvested ipsilaterally to the side with suspicion of one level III metastasis distant from the flap and its pedicle, finally not confirmed by histopathological examination. In summary, all flaps and their pedicles were harvested in the pathologically negative parts of the neck in terms of lymph node metastases or invading SCC. Only in 1 case (no. 4), congruence between the clinical and pathological staging of cervical lymph node status was found out, even by using hybrid imaging methods (fluorine-18 fluorodeoxyglucose PET/computed tomography or PET/magnetic resonance imaging) for clinical staging. This confirms the well-known fact that only histopathological examination can be able to definitively diagnose a metastasis in the lymph node.

The t-FAMMIF-SMG local chimeric flap is a reliable, well-vascularized flap with relatively predictable vascular anatomy. It represents an easily adoptable surgical technique available without microvascular training and preoperative vascular workup. But based on experience with the facial artery myomucosal or SMG free flaps,^30^,^31^ t-FAMMIF-SMG flaps would be probably feasible also as a free flap. For tongue and floor of mouth reconstruction, this flap offers already well-known benefits associated with t-FAMMIF (pliability, thin structure, tissue match, saliva secretion, possible sensation recovery) supplemented with the tissue bulk represented by the SMG for dead space-filling or additional oral lining repair. It may be considered as an alternative for microvascular free tissue transfer in medium-sized oral defects in medically compromised patients. The oncological safety of the t-FAMMIF-SMG local chimeric flap has yet to be verified. This issue may be resolved in patients without risk of neck involvement by tumor or metastases or in a noncancer setting, ideally with necessity or already performed cervical approach. The present study is limited by a small sample of cases, retrospective nature, and short follow-up according to both oncological and functional assessment points of view. Ideally, prospective studies comparing this innovative reconstructive procedure with other options, including the oncological safety evaluation are needed.

## CONCLUSION

Tunnelized facial artery myomucosal island flap and SMG local chimeric flap expands the reconstruction options of medium-sized defects after ablative oral cancer surgery in carefully selected patients primarily not suitable for free flap reconstructions.

## Supplementary Material
