# Supplementary material for: Novel Local Chimeric Flap Based on Tunnelized Facial Artery Myomucosal Island Flap and Submandibular Gland Flap for Reconstructions After Oral Squamous Cell Carcinoma Surgery
Source: J Craniofac Surg. 2022 Aug 9;34(1):76–82. doi: 10.1097/SCS.0000000000008862 (PMC9794126; doi:10.1097/SCS.0000000000008862)
Supplement: Supplementary file 1 [file scs-34-076-s001.docx]

Supplementary Digital Content Table 1. Characteristics of the study patient population.

| **No.** | **Gender/ Age (yrs)** | **Tumor localization** | **cTNM**  **(lymph node involvement suspicion level)** | **Type of ablative surgery** | **t-FAMMIF-SMG local chimeric flap side** | **pTNM** | **Flap-related complications** | **Recipient site complications** | **Donor site complications** | **Adjuvant therapy** | **Oncologic outcomes** | **Follow-up**  **(mths)** |
| --- | --- | --- | --- | --- | --- | --- | --- | --- | --- | --- | --- | --- |
| 1 | M/57 | FOM and tongue ventral surface, left side with contralaterally extension | T3N1M0  (III, left) | Tumor excision with partial glossectomy, sagittal inner table mandibulectomy, ND R (I-III), ND L (Ib-V) | Right | T3N0, G2, L0, Pn0, R0, stage III | None | None | None | RT | NED | 15 |
| 2 | F/75 | FOM and tongue ventral surface, left side | T2N0M0 | Tumor excision with partial glossectomy, sagittal inner table mandibulectomy, ND L (I-III), ND R (Ib) | Right | T3N2b (left), G2, L1, Pn0, ENE-, R0, stage IVa | None | Mandibular fracture, residual tongue partial necrosis | None | RT | NED | 16 |
| 3 | F/73 | Right tongue margin reaching the midline | T3N1M0  (III, right) | Extended hemiglossectomy ND R (I-V), ND L (Ib-III) | Right | T3N0, G1, L0, Pn1, R0, stage III | None | None | None | None | NED | 7 |
| 4 | M/59 | FOM and tongue ventral surface, left side with contralaterally extension | T3N2bM0  (2 LN, III and V, right) | Tumor excision with extended anterior hemiglossectomy ND R (I-V), ND L (Ib-IV) | Left | T2N2b (right), G1, L0, Pn1, ENE -, R0, stage IVa | None | SSI | None | CHRT | NED | 6 |

M, male; F, female; FOM, floor of mouth; TNM staging according to 8th edition of the Union for International Cancer Control TNM classification^16^; LN, lymph node; ND L/R (), left-sided/right-sided neck dissection (levels); t-FAMMIF, tunnelized facial artery myomucosal island flap; SMG, submandibular gland; RT, radiotherapy; CHRT, chemoradiotherapy; SSI, surgical site infection; NED, no evidence of disease.

Supplementary Digital Content Table 2. Functional and aesthetic outcomes after reconstruction with t-FAMMIF-SMG local chimeric flap.

| **No.** | **Mouth opening** | **Oral commissure symmetry** | **Oral intake** |
| --- | --- | --- | --- |
| 1 | 2 | 2 | 2 |
| 2 | 2 | 2 | 2 |
| 3 | 2 | 3 | 1 |
| 4 | 2 | 2 | 1 |

mouth opening (0-3), 0 serious limitation (<0.5 cm), 1 partial limitation (0.5-1.5 cm), 2 minimal limitation (1.5-3.0 cm), 3 no limitation; oral commissure symmetry (0-3), 0 severe downward retraction of oral commissure with severe asymmetry, 1 moderate retraction and asymmetry, 2 mild alteration of symmetry, 3 complete symmetry preservation; oral intake (0-2), 0 – none, full percutaneous endoscopic gastrostomy dependence, 1 partial, perorally soft diet combined with nutrition via gastrostomy, 2 full oral intake
